# Arm Ability Training (AAT) Promotes Dexterity Recovery After a Stroke—a Review of Its Design, Clinical Effectiveness, and the Neurobiology of the Actions

**DOI:** 10.3389/fneur.2018.01082

**Published:** 2018-12-11

**Authors:** Thomas Platz, Martin Lotze

**Affiliations:** ^1^BDH-Klinik Greifswald, Centre for Neurorehabilitation, Intensive and Ventilation Care, Spinal Cord Injury Unit, University of Greifswald, Greifswald, Germany; ^2^Functional Imaging Unit, Center for Diagnostic Radiology, University of Greifswald, Greifswald, Germany

**Keywords:** stroke, arm, training, rehabilitation, plasticity

## Abstract

Arm Ability Training (AAT) has been specifically designed to promote manual dexterity recovery for stroke patients who have mild to moderate arm paresis. The motor control problems that these patients suffer from relate to a lack of efficiency in terms of the sensorimotor integration needed for dexterity. Various sensorimotor arm and hand abilities such as speed of selective movements, the capacity to make precise goal-directed arm movements, coordinated visually guided movements, steadiness, and finger dexterity all contribute to our “dexterity” in daily life. All these abilities are deficient in stroke patients who have mild to moderate paresis causing focal disability. The AAT explicitly and repetitively trains all these sensorimotor abilities at the individual's performance limit with eight different tasks; it further implements various task difficulty levels and integrates augmented feedback in the form of intermittent knowledge of results. The evidence from two randomized controlled trials indicates the clinical effectiveness of the AAT with regard to the promotion of “dexterity” recovery and the reduction of focal disability in stroke patients with mild to moderate arm paresis. In addition, the effects have been shown to be superior to time-equivalent “best conventional therapy.” Further, studies in healthy subjects showed that the AAT induced substantial sensorimotor learning. The observed learning dynamics indicate that different underlying sensorimotor arm and hand abilities are trained. Capacities strengthened by the training can, in part, be used by both arms. Non-invasive brain stimulation experiments and functional magnetic resonance imaging data documented that at an early stage in the training cortical sensorimotor network areas are involved in learning induced by the AAT, yet differentially for the tasks trained. With prolonged training over 2 to 3 weeks, subcortical structures seem to take over. While behavioral similarities in training responses have been observed in healthy volunteers and patients, training-induced functional re-organization in survivors of a subcortical stroke uniquely involved the ipsilesional premotor cortex as an adaptive recruitment of this secondary motor area. Thus, training-induced plasticity in healthy and brain-damaged subjects are not necessarily the same.

## Motor Deficits of Stroke Survivors With Mild to Moderate Arm Paresis

Arm paresis post stroke shows a bi-modal distribution. Many stroke survivors have either severe arm paresis and are only able to use their arms functionally in everyday life to a very limited extent, if at all, or mild to moderate arm paresis with the ability to use their paretic arm for functional tasks, yet with a lack of dexterity (1, 2). Thus, the motor control deficits of these subgroups are quite different and hence so too are their therapeutic needs.

Clinically, stroke survivors with mild to moderate arm paresis have reduced strength and endurance of their paretic arm and are functionally limited by a lack of speed, accuracy and co-ordination of arm, hand, and finger movements and a lack of dexterity when handling objects. Key to understanding any functional deficits and the need and opportunities to improve function by training is a focused analysis of the specific motor control deficits involved in this clinical syndrome. A way to do this is to test various domains of sensorimotor control that have been shown to be independent by factorial analysis (3, 4).

When motor performance of healthy people across various tasks has been analyzed by factorial analysis certain independent arm motor abilities have been documented. These are different independent sensorimotor capacities that together contribute to our skilfulness in everyday life. What are these abilities? They are our ability to make fast selective wrist and finger movements (wrist-finger speed), to manipulate small objects (finger dexterity) or larger objects (manual dexterity) efficiently, our ability to keep our arm steady (steadiness), to move our arm quickly and precisely to an intended target (aiming), or to move it under constant visual control along a line (tracking) (5).

When tested among stroke survivors with mild to moderate arm paresis all these abilities are deficient, indicating the complex nature of sensorimotor control deficits in this clinical condition (6, 7).

## The Arm Ability Training as a “Tailor-Made training” to Meet Specific Rehabilitation Demands

**Figure 1:**
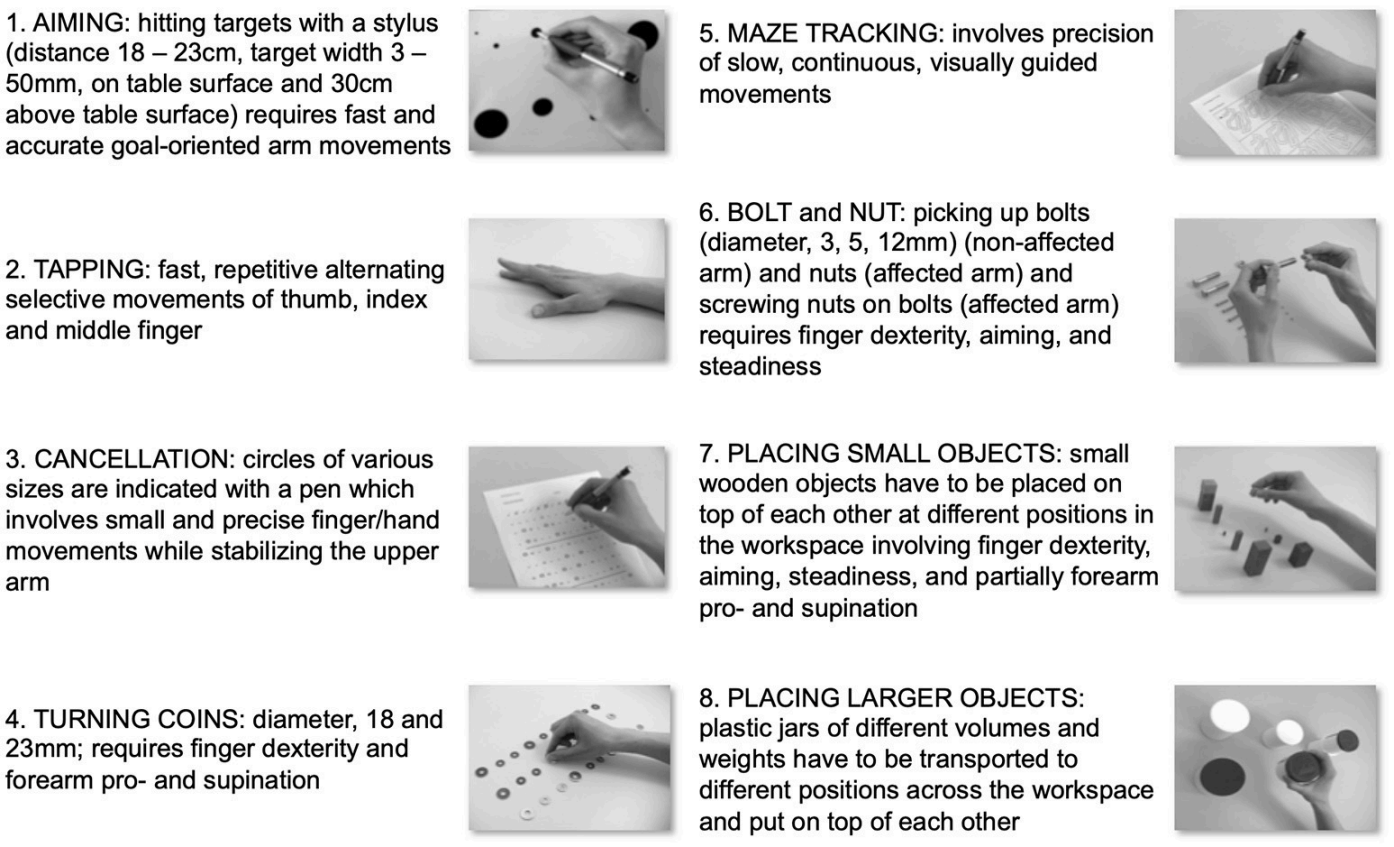
Training tasks of the Arm Ability Training. Description of the eight training tasks of the Arm Ability Training (AAT) that are repetitively exercised daily. Together they train various independent arm and hand sensorimotor abilities. During the AAT sensorimotor performance is trained at its individual limit. Further aspects thought to promote motor learning are a high repetition rate of trained tasks, variation in the difficulty of training tasks, and the augmented feedback provided in the form of intermittent knowledge of the results.

The Arm Ability Training (AAT) was designed to train all these sensorimotor abilities and thus to meet the specific rehabilitation demands of this subgroup of stroke survivors (8, 9). The eight training tasks collectively cover these affordances (Figure 1).

Other factors thought to enhance motor learning were incorporated in the design of the AAT (5):

Repetition: The training has a highly repetitive structure. It has long been known that the establishment of motor skills needs repetition-mediated practice (10).

Variability-of-practice: The different training tasks are each constructed to have a varying task difficulty. Thus, the brain needs to generate and regulate variation in motor control across and within the training tasks, which together with the repeated structure and the explicit intention to improve performance promotes motor learning (11).

Focus of attention, type and distribution of augmented feedback: During the training, patients are encouraged to continuously improve their speed and accuracy, i.e., their performance (as opposed to patterns of joint motion); to promote this emphasis they receive verbal instructions and are intermittently given their results during training sessions (8). This strategy helps focus the attention on the behavioral task as opposed to the movement pattern, and also focuses on the training goal, which is to not only repeat training tasks, but also to improve performance (12).

Overall, the training addresses the motor control deficits both in a targeted way and comprehensively, i.e., across abilities, and does so with a training structure that supports motor learning. Neuroscience knowledge about the specific motor control deficits which characterize this clinical syndrome is thus embedded in the training structure and combined with a high “density” of othertraining aspects that support motor learning and recovery of sensorimotor control for stroke survivors with mild to moderate arm paresis.

A practical description of the AAT has been provided in Platz (13).

## Clinical Effectiveness of the Arm Ability Training

The clinical effectiveness has been tested with two single-blind randomized controlled studies (RCT) (8, 14), one being a multi-center study (14). In addition, the data have been synthesized for stroke survivors in an individual patient data meta-analysis (15).

The first RCT tested the efficacy of the AAT with a sample of traumatic brain injury (TBI) and stroke patients with arm paresis (8). Seventy-four patients were enrolled, 60 (45 stroke patients, 15 with TBI) completed the study; 37 patients were available for an additional 1-year follow-up. During a 3-week intervention period participants received either no AAT (*n* = 20), AAT without knowledge of results (*n* = 20), or AAT with knowledge of results (*n* = 20). The time needed for tasks resembling the arm activities in daily life (Test Evaluant les Membres superieurs des Personnes Agees, TEMPA) (16) and a kinematic analysis of aimed movements were the main outcome measures. After the period of training, the improvement rates were greater among patients who had received the AAT compared to the controls. The mean change score for all TEMPA tasks was 41.4 vs. 12.8 s (*p* = 0.0012), for unilateral TEMPA tasks 16.5 vs. 4.2 seconds (*p* = 0.0036); and for the ballistic component of aimed movements 96 vs. 20 ms (*p* = 0.0115). Whether the AAT was performed with or without knowledge of results had no discernable effect. At the 1-year follow-up there was still an advantage for those who had previously received the AAT. This RCT therefore documented that the AAT provided as “add-on” therapy reduced focal disability associated with mild central arm paresis after TBI and stroke (with a long-term effect).

Further, a multi-center RCT compared the effects of (a.) passive arm and hand splinting with active arm motor training that could be either (b.) individualized best conventional therapy (CONV) or (c.) standardized impairment-oriented training (IOT) in 148 subacute stroke patients with mild to severe arm paresis (14). Participants received 45 min of additional daily arm therapy over 3 to 4 weeks as either (a.), (b.), or (c.). For patients who had severe arm paresis IOT was provided as Arm BASIS training (9), for those who had mild arm paresis it was provided as AAT.

For participants with severe arm paresis the Fugl-Meyer arm motor score (17, 18) testing the selective movement capacity was the primary outcome measure, for participants with mild arm paresis it was the TEMPA time scores (16). Pre–post changes were analyzed to assess the immediate training effects and pre−4 weeks follow-up changes were used to assess any long-term effects. Both per-protocol and intention-to-treat analyses indicated greater improvement rates for participants who had received IOT as treatment compared to best conventional therapy (Fugl-Meyer, arm motor scores: IOT +12.3, CONV +9.2 points; TEMPA: IOT 31.1 s, CONV 20.5 s; *P* = 0.0363); and again, for mildly affected patients, i.e., those receiving the AAT superior long-term effects were shown. Since both of these groups (CONV and IOT) had received the same therapeutic time the specific focus of the active training seemed more important for motor recovery than the intensity (therapy time). The comprehensive modular IOT approach induced motor recovery to a higher degree than best conventional treatment and did so for a broad range of arm paresis post stroke, i.e., from mild to severe arm paresis.

A meta-analysis of individual patient data confirmed the clinical effectiveness of the AAT for stroke patients who have mild to moderate arm paresis, with a greater effect on motor recovery (focal disability) compared to other active motor rehabilitation or none and a moderate differential effect size in favor of AAT (15): Motor recovery (arm/hand function) (standardized mean difference (SMD) for pre-post change scores) in the AAT group was 0.51 standard deviations higher (95% confidence interval (95% CI); 0.11 to 0.91 higher) (*P* = 0.0133; 2 studies, 125 participants).

Both the training's effectiveness and its superiority compared to time-equivalent conventional therapy for stroke patients who have mild to moderate arm paresis are noteworthy and need to be compared to the effectiveness of other arm rehabilitation techniques. Training-based therapies that had been tested in post-stroke arm rehabilitation over the last decades include the constrained-induced movement therapy (CIMT), task-oriented training, bilateral training, mirror therapy, neuromuscular electrical stimulation (NMES) and, arm-robot-therapy. Most of these are clinically useful for stroke survivors with severe arm paresis (i.e., bilateral training, mirror therapy, neuromuscular electrical stimulation (NMES) and arm-robot-therapy), but not for mild to moderate arm paresis like the AAT. The CIMT is useful for stroke survivors with mild to moderate arm paresis, but is indicated for a subgroup who—in spite of their capacities to use their affected arm—do not use their affected arm in daily life or do so only to a rather limited extent. This occurs as a result of learning early on post stroke that the arm will not be useful for daily life activities and consequently a learnt non-use of the affected arm is developed and retained even though the arm might have recovered in the meantime. In this situation CIMT is clinically effective; it can reverse the learnt non-use and induce more and adequate use of the affected arm in daily life (19). The AAT, however, is applicable to stroke survivors with mild to moderate arm paresis independent of the presence of learnt non-use. The therapy which has the biggest overlap with the AAT in terms of the target population is the task-oriented training. Here, the conceptual idea is that when training tasks resemble the activities of daily life, the brain is comprehensively engaged during training, and hence might have adequate stimuli to re-learn arm motor skills. The clinical evidence for task-oriented training in arm rehabilitation post stroke is, however, relatively weak. A large US-based multicentre RCT that randomized 361 subacute stroke patients with moderate arm impairment who then either received 30 h of task-oriented training over 10 weeks, or time-equivalent conventional occupational therapy, or monitored occupational therapy only, did not reveal any statistically or clinically relevant differences between these groups both when assessed after the intervention and during the follow-up up to 12 months post stroke (20). Hence, it was not possible to substantiate an effect of task-oriented training on arm function in this large clinical trial. A Cochrane review documented low-quality evidence that repetitive task training improves arm function (SMD 0.25, 95% CI 0.01 to 0.49; 11 studies, 749 participants) and hand function (SMD 0.25, 95% CI 0.00 to 0.51; eight studies, 619 participants) with a small effect (SMD < 0.40) (21). These comparisons were no longer statistically significant in sensitivity analyses that removed studies with a high or unclear risk of bias for allocation concealment, questioning the stability of the effects when accounting for risk of bias.

But why should task-oriented training be clinically less effective than the AAT? Since no head-to-head comparison is available the evidence needs to be treated with caution and as indirect. Nevertheless, the reason for the observed discrepancy in clinical effectiveness between the two approaches might be that task-orientation is neither a necessary nor a sufficient training characteristic for determining the clinical effectiveness in that patient population. As will be pointed out below, the AAT (which does also systematically involve handling objects) has a variety of characteristics that are not genuine characteristics of a task-oriented training, and which can systematically promote motor learning and motor recovery in stroke survivors. While the evidence thus far shows a clinically relevant lasting effect for subacute stroke survivors with mild to moderate arm paresis receiving AAT, it is also important to report on the limitations of the available evidence. The total number of participants in RCTs testing the AAT's effectiveness is lower than would be needed to enable a precise estimate of its therapeutic effects (i.e., <400). Thus, the estimate of the magnitude of its effect could well change when further trials become available for a meta-analysis. In addition, the reported evidence has been generated during the subacute phase of stroke and inpatient rehabilitation. A low drop-out rate [2% drop-outs among participants randomized to IOT in the biggest multicentre trial (14)] indicates a high acceptability of the IOT interventions tested in that situation. Nevertheless, we do not have evidence for the training's acceptability and effectiveness when applied in the chronic stage and in an ambulatory situation.

## Sensorimotor Learning Induced by the Arm Ability Training

Given the clinical effectiveness of the AAT and its superiority for the therapeutic domain of focal disability recovery after a stroke what are the neurobiological mechanisms of its action?

Motor skill acquisition is generally thought to be dependent on specific repeated practice with an “initial phase” of rapid improvement within single sessions of practice caused by strategic adaptation and behavioral response selection for a motor task and a “slow phase” of gradual improvement with a (true) increase in motor performance, i.e., improved capability of the motor system with an improved speed-accuracy relationship rather than a functional adaptation within the limits of a constant level of performance only (speed-accuracy trade-off) (10).

In healthy subjects motor learning has previously been investigated with various types of motor tasks, e.g., discrete, serial, or continuous tasks and various modifying conditions such as massed vs. distributed practice, different degrees of variability-of-practice, and the type and distribution of feedback given (5). This knowledge has been explicitly embedded in the structure of the AAT and hence it is of interest to assess (physiologically) whether and to what degree motor learning can be achieved with AAT.

We performed a number of studies with the AAT, addressing the behavioral question of its effects on sensorimotor learning in healthy subjects. While the training was designed in such a way as to have a high chance of inducing comprehensive motor learning (within and across abilities), it is worthwhile testing whether it actually achieves this goal. Such knowledge has significant translational significance. A robust induction of motor learning in healthy subjects with measurable effects on dexterity could be a key element in terms of its clinical effectiveness. Stroke survivors with mild to moderate arm paresis, for whom the AAT has been “tailor-made” can use their arms in everyday life, but are less dexterous. Accordingly, their brain is confronted with the same motor control affordances as healthy subjects are and they can cope, but at a lower level of performance than healthy subjects.

While this type of translational research, i.e., the effects of a “therapeutic” training in healthy subjects, has not usually been conducted for other arm rehabilitation therapies, it can be regarded as a relevant research milestone for safeguarding the development of clinically effective therapy.

### The AAT Induces Substantial Sensorimotor Learning

In several experiments it has repeatedly been shown that the AAT promotes considerable and robust sensorimotor learning in healthy right-handed adults training their left non-dominant arm (22–26). As participants in the AAT have to comply with the precision demands, which are made explicit for each of the eight training tasks (Figure 1), any training-induced sensorimotor learning is reflected in the reduced time needed to fulfill work packages given for each training task. When the duration for each work package is standardized for baseline values (= 1.0 at baseline) the time needed to fulfill the work packages after 5 days of daily training (~60 min) has been shown to decrease to 0.79 (95% CI 0.73 to 0.84) (*P* < 0.0001) (14) to 0.72 (95% CI; 0.71 to 0.74) (*P* < 0.0001) (16) on average (across tasks), denoting a 20 to 30% improvement after training. After 10 days an improvement rate of ~30% has been documented (26), and after 16 days ~34% (*P* < 0.0001) (15) which resembles a typical learning curve for motor learning.

Thus, the AAT induced a considerable repetition-mediated increase in performance (speed and accuracy) with incremental gains in motor performance following prolonged daily practice (10).

### The AAT Induces Learning for Different Sensorimotor Abilities

Across the eight different AAT training tasks the improvement rates and thus the learning dynamics were consistently different (22–25) with tapping showing the least improvement; e.g., after 16 days of daily training the improvement in performance ranged from 0.77 (95% CI; 0.72 to 0.81) for tapping to 0.60 (95% CI; 0.51 to 0.69) for nuts and bolts (23), and thus improvement rates varied across AAT tasks from 23 to 40% on average. This is noteworthy since the tasks had been deliberately chosen to collectively address different (independent) sensorimotor abilities and thereby to achieve an effect on sensorimotor performance in daily life which was as broad as possible. Different learning dynamics across tasks do support the assumption that they addressed different control affordances.

To test this notion more specifically, a principal component analysis (PCA) for all behavioral data was performed in one of the experiments (24). Data from the eight AAT tasks, 4 work packages per task and day for 5 days [except for work package 1 on day 1] were used and thus 19 variables per task for 18 participants. The PCA revealed a meaningful 8-component solution and thus a high degree of independence for subsets of variables. The total communality estimate for the model was 123.4, the communalities for each factor ranged from 12.4 to 20.9. In addition, a considerable to high loading of each AAT task on just one (each) of the 8 components was observed.

Taken together, the PCA indicated a high degree of independence of the behavioral data across the arm ability tasks for the repeated measurements taken during the training while the data for each arm ability task during the period of training loaded highly on just one of eight different independent components. This observation further supports the notion that the AAT trains different sensorimotor abilities and might well be a reason for its (superior) clinical effectiveness.

While motor learning research in healthy subjects typically focusses on one of several types of motor tasks, e.g., a discrete, serial, or continuous task (5, 27), here we have proof that the parallel repeated and prolonged practice of different motor tasks both addresses a variety of independent affordances (“abilities”) and induces a substantial improvement in performance level (i.e., motor learning) across these tasks and hence “abilities.” This observation has again significant translational relevance as it shows that the AAT induces the intended *comprehensive* motor learning and thus is a good candidate for assisting the recovery of stroke survivors with mild to moderate arm paresis who have been shown to have performance deficits across these “abilities” (4).

### The AAT Induces Both Limb-Dependent and Limb-Independent Sensorimotor Learning

Another behavioral observation is noteworthy. While only the left arm and hand had been trained in the aforementioned experiments with healthy participants, there was, again consistently (when assessed) a partial transfer of motor learning to the non-trained right hand. This was evident when it was used to perform the AAT tasks after the course of training (compared to baseline assessment) (23, 25, 28). E.g., after 10 days AAT the improvement rate for the trained left hand was 30% (*P* < 0.001) and 19% for the non-trained right hand (*P* < 0.001) indicating a common proportion of 63% for both hands (25). Looking at the pattern of improvement for either hand over 3 weeks (assessed once per week) and for each AAT task separately gave the impression of a qualitatively similar task-specific pattern of improvement for both hands (varying across tasks) with a partial benefit for the non-trained right hand (23). Further, effects of the AAT on the non-trained right hand were not only been observed for the trained AAT tasks, but also when the right hand was assessed with a standard assessment of finger dexterity, the Nine-Hole-Peg-Test, NHPT (29) after a course of AAT (22–24). After 3 weeks (16 days) of AAT the improvement rate for the NHPT were on average 13 and 14% for the left and right hand, respectively, (*P* = 0.0006; effect size d = 0.90) (average time needed for the NHPT: baseline right hand 16.7 s, 95% CI 15.3 to 18.0; left hand 17.4 s, 95% CI 16.2 to 18.6; week 3 right hand 14.3 s, 95% CI 13.3 to 15.3; left hand 15.1 s, 95% CI 14.1 to 16.1) (23).

Taken together, these observations suggest that the AAT trains in part sensorimotor capacities that can be used by either hand, i.e., which are end-effector (limb) independent. Biologically it makes sense that the complex nature of the training not only uses brain processes that are tightly linked to the contralateral sensorimotor system for each limb, but also to activities of brain networks that support sensorimotor control for either limb.

In the literature such a transfer of skill has been shown from a trained to a non-trained finger with a finger tracking task applied over a week (30). And more comparably, a transfer of finger dexterity to the non-trained dominant right hand has also been shown when healthy subjects trained handwriting with their left hand for 15 days (31).

## Training-Induced Plasticity

The behavioral evidence therefore points to a diversity of sensorimotor processes that are improved by this training, i.e., “abilities.” They show training-induced improvements to a varying extent and with a partial transfer of effects to the non-trained arm. Given this knowledge it would be of interest to learn about the involvement and particularly any differential involvement of cerebral regions and network “nodes” during AAT-induced learning for the various abilities and how this might change over time. The significant motor learning that could be behaviourally observed with prolonged repeated practice must be associated with changes in how the brain performs motor control for these tasks (and abilities), i.e., training-induced plasticity.

### Effects of AAT-induced Sensorimotor Learning Can be Modified by Non-invasive Cortical Brain Stimulation

To probe the involvement of sensorimotor cortical areas in AAT-induced motor learning tests were conducted to determine whether AAT-induced learning could be altered by inhibitory or excitatory non-invasive brain stimulation (22–24).

The first experiment used an excitability reducing, inhibitory rTMS protocol (continuous theta burst stimulation with a total of 600 stimuli, cTBS-600) (22). The hypothesis was that at an early stage in the AAT, i.e., during the first few days of training, cortical sensorimotor areas contralateral to the trained arm would be involved in motor learning, and if so, learning dynamics could experimentally be reduced by an inhibitory non-invasive brain stimulation. To test this experimentally seven healthy young subjects trained their left non-dominant arm with the AAT once a day for 5 days. cTBS-600 was applied between the first and second half of individual AAT sessions on four consecutive days (days 2 to 5 of the experiment). With permutation of the order, for each participant cTBS-600 was applied to either the primary motor cortex (M1), supplementary motor area (SMA), premotor cortex (PMC), or primary somatosensory cortex (S1) during one session. The specific objectives were to test whether the motor learning dynamics within sessions could negatively be influenced by cTBS-600, and if so whether the stimulation site (M1, S1, PMC, or SMA) mattered and whether effects were different for the different AAT tasks. The effect on motor learning was analyzed with intra-session effects which examined how cTBS-600 affected motor improvement from the 1st to the 2nd half of each training session. On average these intra-session improvement rates had been in the range of 2%. It was then analyzed whether cTBS modified these rates. For this purpose, effects of cTBS-600 to one site was compared to the data from all other sites. Overall, cTBS-600 to S1 had a more detrimental effect on motor learning than stimulation to the other sites (M1, PMC, S1) (*P* = 0.0432; effect size d = 1.04; absolute differential detrimental effect for cTBS over S1: +0.9%).

This is perhaps not unexpected since the AAT tasks do involve sensorimotor integration. A reverse experimental approach, i.e., peripheral electrical somatosensory stimulation of the fingertips of the trained hand prior to the AAT increased the training gain achieved over 10 days by 3.4% (on average 32.9% improvement rate with somatosensory priming vs. 29.5% without) (*P* = 0.044; effect size d = 0.77) (26).

Back to the cTBS-experiment (22): There were also effects that were specific for individual AAT tasks. Tapping was most affected by cTBS-600 to M1 (*P* = 0.0341; effect size d = 2.73) and aiming as well as placing large objects by cTBS-600 to PMC (*P* = 0.0249; effect sizes d = 2.97 and *P* = 0.0249; d = 1.21, resp.). Thus, improvement of fast isolated finger movements was most affected by cTBS-600 to M1, while any improvement of motor behavior that involved the navigation of the arm in extrapersonal space was most affected by cTBS-600 to PMC.

The experiment was repeated with twelve right-handed AAT-naive volunteers who, however, had a prolonged course of 3 weeks AAT (instead of 5 days). In this experiment, when the same cTBS-600 applications were used in the final 3rd week of training, no cTBS-effects on learning dynamics within the session could be corroborated (23).

Overall, the results of these two experiments would suggest that motor learning with the AAT involves the cortical areas S1, M1, and PMC critically during the first days of training and much less so after extended training over a few weeks.

This observation is in line with both animal and human data assessing cortical involvement during prolonged motor learning. Picard et al. (32) documented a decreased activation of the primary motor cortex (M1) in monkeys when performing highly over-trained internally-generated sequences of reaching movements most likely indicating a higher synaptic efficacy achieved by prolonged training. Wymbs and Grafton (33) observed an overall and skill-specific decrease in the contralateral primary sensorimotor cortex and premotor regions including PMC and SMA as well as the posterior parietal cortex with prolonged finger sequence learning.

Thinking along these lines, it was also of interest to find out whether the reverse intervention, i.e., an excitatory non-invasive brain stimulation to one of these areas could enhance the AAT-induced training effect. For this purpose, eighteen healthy young subjects trained their non-dominant left arm with the AAT once a day for 5 days using (24). In this case an excitatory form of rTMS, i.e., intermittent theta burst stimulation, iTBS-600 was used for priming purposes. Participants were assigned to three groups that received either (A) sham stimulation with a placebo-coil to the right M1, (B) iTBS-600 to the right M1, or (C) iTBS-600 to the right S1 on days 2 to 5 of the experiment. There was a numerically small, yet statistically significant difference in favor of subjects who received iTBS compared to sham stimulation for 4 days directly prior to the AAT: The final level of performance at the end of training was on average 0.72 across the arm ability tasks for the group receiving iTBS compared to 0.74 for the sham group (*P* = 0.0285), indicating a differential benefit in improvement rate of 2%. Differences between iTBS to either M1 or S1 could not be corroborated statistically in this experiment. There was, however, a generalization of the effect of iTBS on motor learning to the non-trained finger dexterity task, the NHPT, specifically for the trained left hand (*P* = 0.0414). The pre-post improvement was 13% on average for the left hand NHPT performance among those receiving iTBS, but only 7% among those receiving sham stimulation.

Thus, priming with iTBS-600 to either M1 or S1 enhanced motor learning during the AAT.

In the literature, the effects of non-invasive brain stimulation have been variable (34). The reported observations are nevertheless consistent and in agreement with experiments with single motor learning sessions where iTBS priming was associated with enhanced motor learning, e.g., for ballistic thumb movements (35) and dexterity after complex training involving a dexterity task, tapping, tracking and a pegboard task (36).

### AAT-induced Changes in Cerebral Activation During Motor Tasks

The behavioral evidence that (a.) substantial sensorimotor learning is induced by the AAT, (b.) the fact that different sensorimotor abilities are addressed and their capacities enhanced by the training, and (c.) the evidence from both inhibitory and excitatory non-invasive brain stimulation experiments that different cortical areas are involved in sensorimotor learning early during the AAT (first week of daily training), but presumably less so at later stages (e.g., after a couple of weeks of training) all require functional imaging data to elucidate the cerebral activation patterns associated with (sensori)motor tasks and their evolution after a course of AAT, i.e., training-induced plasticity.

### Cerebral Activation Patterns Before and After AAT in Healthy Volunteers

For this purpose, fifteen healthy subjects trained their non-dominant left arm for 2 weeks (11 training sessions) with the AAT (25). Functional magnetic resonance imaging, fMRI was performed with three non-trained motor tasks that were performed with both of the participants' hands, i.e., both the trained left and also the non-trained right hand: (A) Participants had to clench their fist with a strength of 33% of their maximum voluntary contraction at a rate of 1 Hz. (B) A finger sequence task with 12 responses of the index, middle, ring, or small finger was performed according to a visual presentation of numbers (corresponding to the digits, i.e., 2 to 5), again with a frequency of 1 Hz. (C) Writing involved copying 10 words in cursive handwriting; individually, the same writing speed was used at pre and post test fMRI.

MRI data were collected before and after 2 weeks of AAT for 14 participants using a 3 Tesla MRI scanner (Magnetom Verio, Siemens, Erlangen, Germany) equipped with a 32-channel headcoil.

After the training period there was a substantial improvement in performance for the AAT tasks (~30% on average) (*P* < 0.001) with a partial transfer to the non-trained right arm (~19% on average) (*P* < 0.001). In addition, the motor tasks used for fMRI also showed improved performance, for both hands in the case of the finger sequence task and for the (trained) left hand with regard to the writing task.

Regarding the fMRI data, only statistically corroborated effects are described here (without individual *P*-values). Activation maps for the main effects and conjunctions were FWE-corrected over the whole brain; training effects were analyzed with a region of interest approach, corrected for multiple comparisons, FWE-corrected.

A decrease in activation when the trained arm was used after the training was observed for the finger sequence tasks in the ipsilateral S1 (BA1, 2, 3), bilateral SMA (BA 6), ipsilateral superior parietal lobe (SPL; BA 5 and BA 7) and inferior parietal sulcus (IPS, BA 40) as well as the dorsolateral prefrontal cortex (dlPFC, Brodman area, BA 8). For the writing tasks a decrease was found in the ipsilateral posterior cerebellar hemisphere (Larsell H VIIA; crus 1).

**Figure 2:**
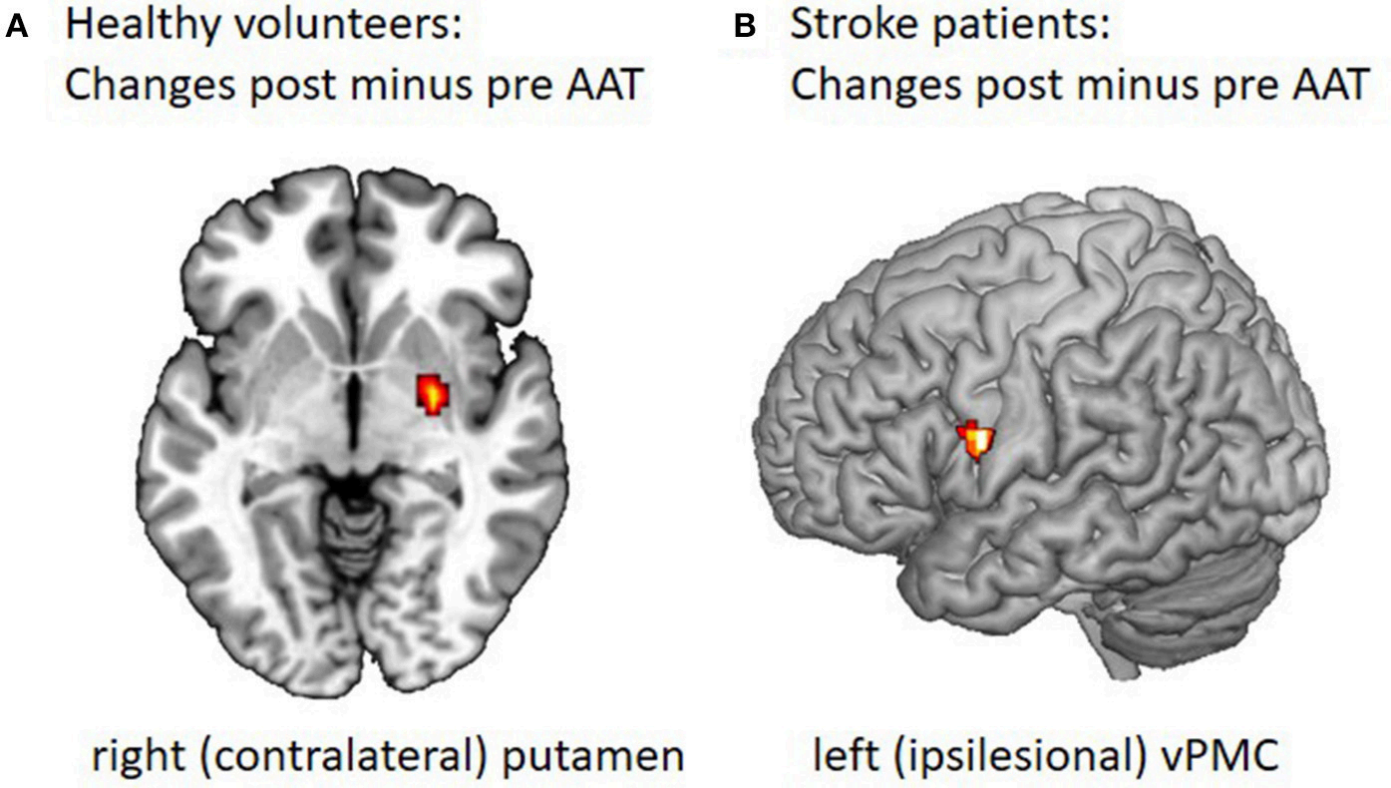
Changes in motor task-related cerebral activation after the Arm Ability Training. **(A)** FMRI data were collected before and after 2 weeks of AAT for 14 healthy participants using a 3 Tesla MRI scanner (25). For the fist clenching task participants were trained to press the ball with the target force being 33% of maximal voluntary and a target rate of 1 Hz. The post minus pre comparison showed increased contralateral putamen and pallidum and ipsilateral putamen activation for fist clenching after training. The ipsilateral putamen activation is to be found at another z-direction height (here z = 0). **(B)** Twelve patients in the subacute phase from 2 to 9 weeks after a mild to moderate motor stroke were recruited for a combined AAT (15 one hour sessions over 3 weeks) and fMRI study during their inpatient rehabilitation stay (37). FMRI during an active and a passive motor task for the affected and unaffected hand was performed before and after a 3 week course of AAT during inpatient rehabilitation. The figure shows the change in activity over time observed in vPMC as measured for the active hand grip task when performed with the affected hand, post minus pre contrast, displayed on a segmented template (Collins Brain).

An increase in activation when the trained arm was used after the training was found in the contralateral pallidum and bilateral putamen for the fist clenching task (see Figure 2A) and the left anterior cerebellar hemisphere (Larsell H VI) for the writing task.

For the non-trained right hand, a decrease in activation during the finger sequence task was documented in the ipsilateral in SPL (BA 5), contralateral cerebellar hemisphere (Larsell H VI and VIIA) as well as the ipsilateral cerebellar vermis (VI and VIIIA).

In summary, after 2 weeks of AAT with the non-dominant left arm the fMRI data showed complex changes in motor-task related activation, i.e., a reduced activation in ipsilateral S1, parietal cortical areas, the SMA bilaterally, and the dlPFC with a finger sequence task, and the lateral cerebellar hemisphere with the writing task. An increase of activation in the striatum was found for the fist clenching task and the anterior cerebellum for the writing task. Thus, cortical motor and non-motor area activation decreased over time, whereas cerebellar anterior hemisphere and striatum activity became more prominent after prolonged sensorimotor training with the AAT. Further, related to the different sensorimotor affordances of the motor tasks used in the scanner, changes in brain network activations were different. These observations are in good agreement with the notion that the AAT induces learning for different sensorimotor abilities and hence training-induced plasticity in the cerebral networks providing these capacities.

Overall, the data is consistent with known cortical-basal ganglia-cerebellar networks involved in human motor learning (38) and corresponding knowledge about motor control, motor skill acquisition and related neuroplasticity from animal studies (27, 39). Previous observations of human motor learning, likewise, showed more prominent cortical involvement in early motor learning and a gradual shift of acquired skill representation to more subcortical activity in motor cortico-striatal and cortico-cerebellar networks with more prolonged training (33, 40, 41). The increase in activation in the striatum with the fist clenching task might mirror an aspect of reinforcement learning by the “sensorimotor” striatum, while the increased cerebellar hemisphere with improved writing for the trained left arm presumably indicates learning based on error signals (27).

For the translational research aspect it is important to note that the complex parallel motor training schedule with the AAT shows typical previously known aspects of the neurobiology of motor learning, i.e., training-induced cerebral plasticity. Furthermore, training-induced brain plasticity manifested itself even with motor tasks that had not been trained, validating the notion that when training tasks address “abilities” comprehensively, motor learning generalizes to untrained tasks (that draw on the same underlying set of “abilities”) and hence change their cerebral activation pattern accordingly.

### Cerebral Activation Patterns Before and After AAT in Subcortical Stroke Patients

Given the cerebral re-organization associated with the AAT in healthy volunteers it is of interest to know whether these changes could likewise be observed in stroke survivors who have survived a subcortical stroke leaving them with a mild to moderate arm paresis and who then receive a 3 week course of AAT.

For this purpose, 12 stroke survivors with mild to moderate are paresis were recruited for a combined AAT (15 one hour sessions over 3 weeks) and fMRI study during their inpatient rehabilitation stay in the subacute stage of recovery (37). Improvement in performance after the training was assessed with both the AAT tasks and conventional hand motor tests [NHPT, Box-and-Block Test, BBT (42)]. The AAT (combined with other rehabilitation efforts) resulted in considerable performance improvements, both in the trained tasks (mean execution time for AAT tasks was reduced on average by 27.7%, *P* < 0.001), and other hand motor functions (NHPT: average time needed to perform the NHPT decreased by 25.2%, *P* < 0.007; BBT: number of blocks moved in the BBT increased by 20.9% on average, *P* < 0.001).

For the parallel fMRI investigation there was a non-trained active and also a passive task used. The active task was a fist clenching task; performance at pre and post-test was kept constant in terms of both force and frequency. For the passive movement task, wrist flexion-extension movements were performed at a rate of 1 Hz with a pneumatically driven splint.

Statistical note for the fMRI data analyses: For the region of interest analyses M1, S1, SMA, dorsal PMC, ventral PMC, and the cerebellum on either side were included. Only statistically corroborated effects corrected for multiple comparisons [p(FWE) < 0.05] are described here (without individual *P*-values).

The active tasks performed with the non-affected hand activated M1, SMA, dPMC, vPMC, S1 and cerebellum in both hemispheres both in the pre-test and post-test. When performed with the affected hand, M1, SMA, dPMC, S1, and cerebellum were activated before the training while there was an additional bilateral vPMC activation after the training. The analysis of longitudinal fMRI changes corroborated a specific effect for the affected hand and the active task only: there was an increase of vPMC activation over time (see Figure 2B). Furthermore, only the analysis for the affected hand performing the active task post training indicated a stronger bilateral vPMC activation than healthy subjects.

These results point toward a need for additional recruitment of this secondary motor network area for survivors of subcortical strokes with mild to moderate arm paresis, in order to improve their performance levels.

In general, our knowledge about training-induced cerebral re-organization for stroke survivors with arm paresis is still limited. A small cohort study with 12 acute subcortical stroke patients with moderate to severe hand paresis who received very early mobilization and task-oriented physical therapy indicated that hand motor recovery was associated with a highly lateralised ispilesional primary sensorimotor cortex activation on fMRI (43). In a substudy of two multi-centere clinical trials with chronic stroke survivors receiving either robotic or intensive conventional therapy for 6 to 12 weeks resting state connectivity was responsive to treatment, showing an increase in affected primary motor cortical connectivity to other frontal motor areas (44). In another small cohort study (10 chronic stroke participants) mental practice over 10 weeks induced both motor performance improvements for the affected hand and associated increases in bilateral primary sensorimotor and premotor activation (45). A systematic review (8 studies, 164 participants) addressing the question of whether bilateral arm training is associated with training-induced plasticity in stroke patients could, however, not corroborate a consistent pattern of cerebral re-organization (46). These examples indicate that training-related changes in the cortical activation and connectivity pattern had been reported for primary sensorimotor and secondary motor areas, yet had been variable. Reasons for this heterogeneity might be that the participants included had been diverse in terms of their stroke characteristics or level of impairment or that the interventions themselves and their effects had been variable. In our study (37) a selective group of patients,who were subacute subcortical stroke survivors with mild to moderate paresis, received a standardized training and showed both clinically relevant motor recovery and an increase in vPMC activation during movements of the affected hand post training. In that constellation vPMC seemed to be an important network node for motor recovery.

## Conclusions

The AAT has been specifically designed to meet the needs of subjects with acquired brain injury with mild to moderate arm paresis and a lack of “dexterity” in everyday life causing focal disability. It explicitly addresses the lack of sensorimotor efficiency across various arm abilities as documented for this subgroup of stroke survivors by (a.) training these abilities, (b.) training sensorimotor performance at the individual limit as well as further aspects known to promote motor learning, i.e., (c.) a high repetition rate of trained tasks, (d.) variation in the difficulty of the training tasks, and (e.) augmented feedback provided in the form of intermittent knowledge of results. The inherent external focus of attention created by the constant drive for improved performance (speed and accuracy) beyond the current individual limit as opposed to a self-centered, non-ambitious “quality of movement”-focus, together with the intermittent provision of results, the almost uniformly experienced improvement of performance within training sessions and from day to day and hence the enhanced expectancy for further success are all likely to direct attention to the task and the training goal and enhance goal-action coupling during the AAT (12). These aspects are strong facilitators for the repetition-mediated improvement of performance, i.e., motor learning (10). The implemented constant change of affordances within tasks (variability of task difficulty) and the shift between tasks which train different sensorimotor abilities is a learning experience for the brain with a high need to generate and increasingly efficiently regulate motor variability, a situation that makes motor skill acquisition highly likely (11).

The clinical effectiveness for stroke patients in the subacute phase with mild to moderate arm paresis was able to be shown by two fairly large single blind randomized controlled trials: The AAT improved sensorimotor efficiency with ADL-like arm activities with a long-term effect. And, it has been shown to be superior to therapeutic time-equivalent “best conventional” therapy.

With regard to the neurobiology of the training, behavioral training data from experiments with healthy subjects training their non-dominant left arm with the AAT over a period of 1 to 3 weeks confirmed that the AAT induces substantial motor learning, with generalization to a non-trained dexterity task and partial transfer to the non-trained arm indicating partially endeffector-independent resources that were improved by the training. The data further supports the notion that the AAT induces motor learning across different independent sensorimotor abilities, a fact that may well be of paramount importance for its clinical effectiveness. Motor learning processes induced by the AAT involved distributed cortico-subcortical networks with task-specific variation of the relevance of cortical areas (as shown for M1, PMC and S1) and a change with less cortical involvement and more basal ganglia and cerebellar activation after prolonged training indicating a consolidation process. All these findings contribute to our understanding of how the substantial increase in motor performance is induced by the training.

It could be shown that the ipsilesional vPMC played a specific role in training-induced plasticity and recovery for patients with subcortical stroke in the subacute phase with mild to moderate arm paresis who received the AAT for 3 weeks and showed a substantial clinical improvement.

In summary, the AAT is a complex motor training tailor-made for stroke survivors with mild to moderate arm paresis; it is intentionally designed to contain a “high density” of aspects that are relevant for achieving substantial training-induced recovery from focal disability. Its structure supports intrinsic motivation and a focus on motor learning and effectively strengthens various sensorimotor arm and hand abilities with long-term effects. The training-induced recovery of arm abilities is based on functional re-organization of related cortico-subcortical cerebral networks with a partially bilateral organization and an adaptive recruitment of the ipsilesional ventral premotor cortex among stroke survivors.

## Author Contributions

TP designed and wrote the manuscript. ML critically revised it for intellectual content and clarity of presentation.

### Conflict of Interest Statement

TP received grants from the German ministry for education and research (BMBF) for clinical trials assessing the effectiveness of the AAT, provides AAT teaching courses for therapists and training material. ML and TP received jointly received grants from the German research community (DFG) for the rTMS and fMRI experiments cited. Thus, both authors have a personal link to the work presented.
